# The hyoid arch and braincase anatomy of *Acanthodes* support chondrichthyan affinity of ‘acanthodians’

**DOI:** 10.1098/rspb.2015.2210

**Published:** 2015-12-22

**Authors:** Martin D. Brazeau, Valerie de Winter

**Affiliations:** 1 Naturalis Biodiversity Centre, 9517, 2300 RA, Leiden, The Netherlands; 2 Institute of Biology, Leiden University, PO Box 9505, 2300 RA, Leiden, The Netherlands

**Keywords:** Palaeozoic, gnathostome phylogeny, synapomorphy, computed tomography

## Abstract

Solving the evolutionary relationships of the acanthodians is one of the key problems in reconstructing ancestral anatomical conditions for the jawed vertebrates (gnathostomes). Current debate concerns whether acanthodians are an assemblage of stem chondrichthyans, or a more generalized grade encompassing some early stem osteichthyans. The skull anatomy of *Acanthodes bronni* has been pivotal in these debates, owing to tension between chondrichthyan- and osteichthyan-like models of reconstruction. We use computed tomography scanning and traditional palaeontological techniques to resolve the long-standing debate about the anatomy of the jaw suspension. We establish the correct length of the hyomandibula and show that it attaches to a process on the ventrolateral angle of the braincase below the jugular vein groove. This condition corresponds precisely to that in chondrichthyans. This character represents an unambiguously optimized synapomorphy with chondrichthyans given current gnathostome phylogenies, corroborating the growing consensus of the chondrichthyan affinity of acanthodians.

## 1. Introduction

The origin of modern gnathostomes (jawed vertebrates) remains one of the most obscure episodes in vertebrate evolutionary history. While there is a wealth of early gnathostome fossils from the Palaeozoic Era, considerable phylogenetic uncertainty leaves the evolutionary significance of these fossils in dispute. In the past decade, significant effort has been spent on reconstructing the phylogenetic relationships of early Palaeozoic gnathostomes in the hope of breaking through this impasse. A core debate has concerned the affinity of the enigmatic ‘acanthodians’: a diverse, possibly paraphyletic, assemblage of Silurian to Permian aged fishes with spine-bearing paired and median fins. Acanthodians combine characters thought to be diagnostic of the two principal lineages of living jawed vertebrates: chondrichthyans (sharks, rays and chimaeroids) and osteichthyans (bony fishes and tetrapods). This character distribution and uncertain phylogenetic position presents a major dilemma in reconstructing the primitive skeletal conditions of early gnathostomes.

An endoskeleton is universal among vertebrates, providing broadly comparable characters that help resolve deep splits in gnathostome phylogeny. Substantial fossil remains of an acanthodian cranial endoskeleton (endocranium: braincase or neurocranium, jaws and visceral/branchial cartilages) are known only from *Acanthodes bronni*. This taxon is well known from three-dimensional fossils preserved in siderite concretions from the Permian of Lebach, Germany. Despite its late geological age and apparent anatomical specializations, *Acanthodes* has served as an endoskeletal proxy for all acanthodians for many decades [1–6].

Until recently, the most complete reconstructions of the skull of *Acanthodes* were those by Miles [3] and Jarvik [6]. The disagreement between these two reconstructions would encapsulate the subsequent debate on acanthodian affinities. One of the central points of debate between Miles and Jarvik was the location of the hyomandibular articulation on the otic capsule sidewall relative to the jugular vein—a key point of demarcation between osteichthyans and chondrichthyans. Miles reconstructed *Acanthodes* with overall osteichthyan-like braincase geometry and placed the hyoid articulation dorsal to the jugular vein groove. Jarvik disagreed with Miles's interpretations, considering *Acanthodes* generally chondrichthyan-like, placing the articulation below the jugular groove. This character is particularly valuable given that there is a logically applicable outgroup condition observed in arthrodire (and probably other) placoderms (electronic supplementary material, figure S1). Here, the hyomandibula is supported on a lateral buttress over the jugular vein (see arguments by [7]), as in osteichthyans and *Janusiscus*, suggesting the chondrichthyan-like condition would be apomorphic.

An osteichthyan placement for *Acanthodes* prevailed largely due to Miles' explicitly cladistic criteria and later Maisey's [4] analysis. However, Brazeau's description of a partial braincase of an Early Devonian acanthodian, *Ptomacanthus*, showed that endocranial anatomy of acanthodians was more diverse than the typology based on *Acanthodes* [8]. The braincase of *Ptomacanthus* is proportioned very differently from *Acanthodes*, resembling chondrichthyans and some placoderms. Brazeau explained the differences between them as arising from acanthodian paraphyly: some acanthodians branching from the osteichthyan stem (such as *Acanthodes*) and others branching from either the chondrichthyan or gnathostome stem (such as *Ptomacanthus*).

Most recently, Davis *et al*. [9] reanalysed the braincase of *Acanthodes*. They identified (or re-identified) a number of chondrichthyan-like characters. Furthermore, they noted fatal problems in the hyoid articulation proposed by Miles, but nevertheless placed the articulation above the jugular vein groove but in a more posterior location, apparently recalling the conditions in sharks. Despite this, the phylogenetic analysis by Davis *et al*. largely echoed Brazeau's in the placement of *Acanthodes*. Not until the discovery of *Entelognathus* [10], a placoderm-grade fish with osteichthyan-like facial jaw bones, did any numerical analyses place the totality of acanthodians on the chondrichthyan stem. This has been corroborated by an array of subsequent studies [7,11,12]. However, these studies derive from the same original dataset and are not entirely independent [13] and no new acanthodian-related data were added.

In this paper, we reassess the reconstruction proposed by Davis *et al*. based on an examination of the same fossils and undescribed specimens, and provide new character evidence corroborating the chondrichthyan affinity of acanthodians. We reveal problems in both Miles' and Davis *et al*.'s reconstructions and reconcile the differences between Jarvik's, Miles' and Davis *et al*.'s interpretations. Our new reconstruction confirms shared chondrichthyan conditions, and corroborates the phylogenetic placement of *Acanthodes* on the chondrichthyan stem; this adds further detail to the transformed perspective on the earliest osteichthyans as more morphologically conservative than traditionally assumed. The unusual braincase of *Acanthodes* is intermediate in structure between osteichthyans and chondrichthyans. However, some of the peculiarities can be explained as adaptations accommodating a large gape, consistent with the inferred suspension feeding ecology of *Acanthodes*.

## 2. Material and methods

### (a) Specimens

This investigation used an unprepared nodule containing the skull of *Acanthodes* from the Museum für Naturkunde, Berlin MfN.f.14117 for CT investigations; a silicone peel of MfN.f. 14089, the same specimen used in the investigation of Davis *et al*. (cited therein as HU MB 3b); and one cast of a dorsoventrally compressed, articulated skull from the Natural History Museum, London NHM P.34914.

### (b) Computed tomography

MfN.f.14117 was scanned in a Phoenix X-ray 180 kV CT scanner at the Museum für Naturkunde, Berlin, using 1440 projections with a magnification of 1.66666695, a voltage of 110 kV and a current of 110 µA, resulting in an initial slice thickness of 0.0599 mm. The nodule was too big for the available field of view in the scanner, so two partially overlapping scan series were generated. The resulting tomography data were exported as TIFF images and stacked together in Fiji [14].

The resulting image series was imported into Mimics, v. 15.01, (Materialise Software) for segmentation and three-dimensional (3D) modelling. We used manual segmentation with the threshold edit to construct mask-based 3D models. Final publication-ready images of the 3D models were rendered using the open-source animation software Blender 3D (https://www.blender.org).

### (c) Phylogenetic analysis

We updated the phylogenetic data matrix from Giles *et al*. [7] with one new character and a coding change derived from this work (electronic supplementary material, data). Phylogenetic analysis was conducted using PAUP* [15]. Two analyses were conducted, one using identical parameters and search procedures to Giles *et al*. [7]; the second using all the same parameters, but conducting a search under implied weighting [16] with a default concavity parameter *K* = 2 and using a rearrangement limit of 50 million per addition sequence replicate.

## 3. Results

### (a) Specimen descriptions

**Figure 1.**
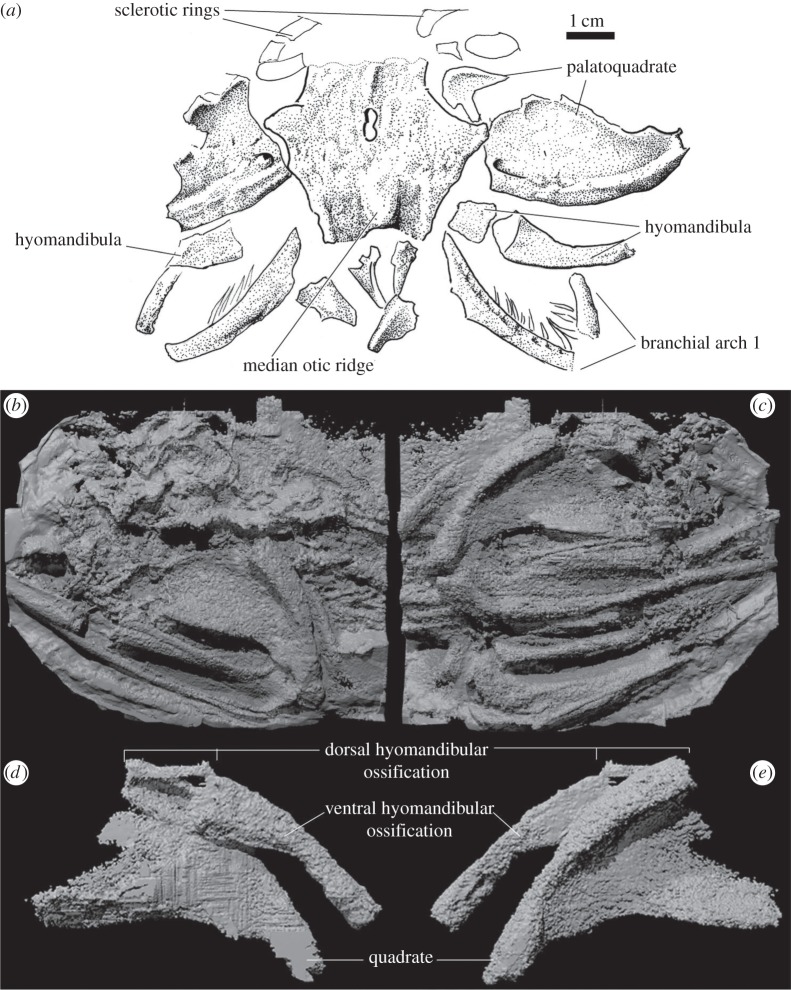
*Acanthodes bronni* specimens demonstrating position of hyoid articulation and length. (*a*) NHM P.34914 showing anteroposterior level of hyomandibular attachment to otic sidewall. (*b*–*e*) Virtual three-dimensional tomography renderings of MfN.f. 14117. (*b*,*c*) Whole specimen in left and right lateral views, respectively. (*d,e*) Isolated right hyomandibula and quadrate ossifications in media and lateral views, respectively.

NHM P.34914 is a dorsoventrally compressed skull of *Acanthodes*, consisting of the dorsal neurocranial ossification, paired lateral occipital plates, paired palatoquadrates, paired hyomandibulae and at least four dorsal branchial arch ossifications (figure 1). As the specimen is dorsoventrally compressed, significant anteroposterior displacement of the elements is not anticipated here. This specimen displays two important details bearing on the reconstruction of the hyoid arch. Firstly, it is evident that the articulation with the otic capsule wall is at an approximate midpoint along its antero-posterior length, contrary to the reconstruction by Davis *et al*. Secondly, we see that proximal (or anterior) and distal (or posterior) ossifications of the hyomandibula are in contact. The proximal end of the distal ossification is embayed in lateral view, and does not contact the proximal ossification. The proximal margin is thus ‘cut’ obliquely across the axis of the hyomandibula, possibly explaining the apparent unossified gap seen in some specimens.

Our examination of MfN.f.14089 corroborates the interpretation of the ventral otic capsule of Davis *et al*. Of particular relevance here is the ‘anterolateral otic process’ of their study. This structure is clearly bounded posteriorly by a groove that may have carried the glossopharyngeal nerve (N.IX).

### (b) Computed tomography

Specimen MfN.f.14117 consists of the mandibular arch, hyoid arch, a small portion of the braincase and branchial arches in the posterior part of the fossil (figure 1*b*,*c*). The mandibular arch is preserved as a quadrate and Meckelian cartilage in articulation (figure 1*b*,*e*). Both hyomandibulae are visible and they are in association with the quadrate. The anterior ossification of the right hyomandibula is thin, and poorly ossified, but present in articulation with the posterior ossification (figure 1*d*,*e*).

### (c) Phylogenetic analysis

The unweighted search returned a result identical in length and tree number to Giles *et al*.: 522 936 trees of length 640 steps. Searching under implied weights returned 216 trees of score −171.00260. As in the most recent analyses of gnathostome interrelationships [7,10–12], acanthodians are recovered in both strict consensus trees (electronic supplementary material, figure S2) as an array of stem chondrichthyans. Implied weighting resulted in greater resolution among stem chondrichthyans. Although acanthodians remain paraphyletic, in both analyses the acanthodiforms, ischnacanthids and diplacanthids form a clade that is itself the sister group of all other total-group chondrichthyans.

## 4. Discussion

### (a) Anatomical critique of Miles' and Davis *et al*.’s reconstructions

Both Miles and Davis *et al*. propose that the articulation of the hyomandibula in *Acanthodes* is dorsal to the jugular groove in a condition deemed osteichthyan-like. Neither study identified a clear articulation facet and effectively had to work by process of elimination. Miles placed the articulation in an anterior location, just behind the trigeminofacial opening. However, Davis *et al*. effectively showed that there is no corresponding facet or even sufficient area on the otic sidewall in this location to accommodate the proximal end of the hyomandibula. Based on their reconstruction of the hyomandibula, Davis *et al*. placed the articulation instead on the posterolateral angle of the braincase, as in chondrichthyans.

Two problems arise from these arrangements. The first is that both Miles and Davis *et al*. place the articulation of the hyomandibula dorsal to both the jugular groove *and* the lateral otic ridge. The latter structure is a superficial signature of the horizontal semicircular canal (electronic supplementary material, figure S1). Furthermore, the placement is posterior to the exits for branches of the glossopharyngeal nerve—a condition unlike any vertebrate, where a fairly consistent order is preserved between visceral arches and cranial nerves. Thus the placement is not osteichthyan-like, but is instead anatomically anomalous (electronic supplementary material, figure S1). Secondly, NHM P.34914 shows that the hyomandibula does not articulate with the posterolateral angle of the braincase but instead along its sidewall. Therefore, we deduce that the position of the articulation must be somewhere below the lateral otic ridge, but anterior to the posterolateral angle of the braincase.

### (b) Alternative reconstruction of *Acanthodes*

**Figure 2.**
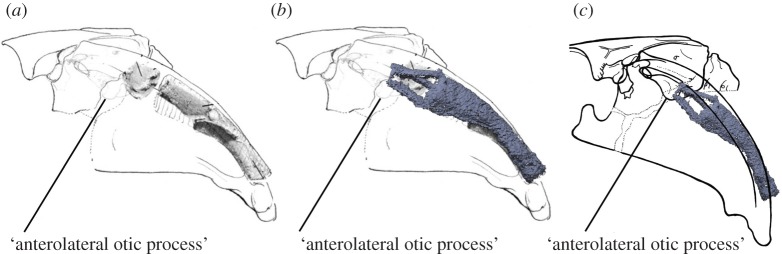
Hyomandibulae superimposed on articulated braincase and palatoquadrate. (*a*) Original restoration by Davis *et al*. [9]. (*b*) Original restoration from [9] with hyomandibula from this study superimposed. This shows the relatively more ventral position of the articulation compared with *a*. (*c*) Reconstruction with palatoquadrate restored to angle used in final reconstruction of [9] and new hyomandibula superimposed in place. This shows the estimated site of articulation near the ‘anterolateral otic process’. (*a*,*b*) Re-used with permission from the author. (Online version in colour.)

Superimposing the articulated hyomandibula of MfN.f.14117 onto the reconstruction by Davis *et al*. results in a different placement for its articulation with the otic capsule consistent with the new observations presented here (figure 2). The antero-posterior coordinate is at mid-length along the otic capsule, in agreement with NHM P.34914. However, the proximal endpoints below the jugular vein groove, to the structure termed the ‘anterolateral otic process’ by Davis *et al*.

**Figure 3.**
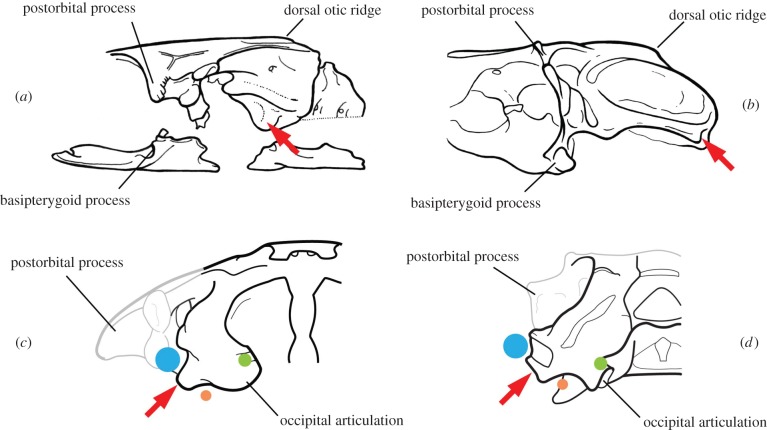
Comparative analysis of the ‘anterolateral otic process’ of *Acanthodes* and the hyomandibular articulation of a chondrichthyan (*Pucapampella*, adapted from [18]). (*a*,*c*) *Acanthodes bronni* in (*a*) lateral and (*c*) posterior views. (*b*,*d*) *Pucapampella* in (*b*) lateral and (*d*) posterior views. Red arrow indicates location of hyomandibular articulation. Blue disc: jugular vein; orange disc: glossopharyngeal nerve exit (N.IX); green disc: vagus nerve exit (N.X).

As noted by Davis *et al*., the ‘anterolateral otic process’ is bounded posteriorly by the groove for the glossopharyngeal nerve (N. IX). Furthermore, this process houses the posterior ampulla of the skeletal labyrinth, which is found in close proximity to the hyomandibular articulation in some early chondrichthyans [17]. The relationship to the glossopharyngeal nerve prompted Davis *et al*. to propose homology of this process with the posterior postorbital process of placoderms. However, we propose here that it corresponds to the lateral otic processes of chondrichthyans. Figure 3 and electronic supplementary material, figure S1 show the anatomical correspondence between these ‘landmarks’ with the proposed location hyoid articulation of the early chondrichthyan *Pucapampella* [18]. Although there could be some uncertainty of placement of the hyomandibula in *Pucapampella*, the inferred position corresponds anatomically with other early chondrichthyans (electronic supplementary material, figure S1) and the position of the articulation in the South African *Pucapampella*-like form [19].

### (c) Phylogenetic implications

Many proposed chondrichthyan cranial apomorphies are difficult to polarize because they are either ambiguous in outgroups (e.g. paired canals in parachordals for the dorsal aorta) or may be correlated with the absence of a macromeric dermal skull roof (e.g. the median otic ridge) [13]. Those that are unambiguous are either not general enough to include *Acanthodes* or the relevant areas are missing in this taxon. However, the site and nature of the hyoid articulation on the otic capsule below the jugular vein is clearly distinguished from a consistent outgroup condition. The site of articulation of the hyomandibula of *Acanthodes* is therefore of particular importance and has been a major point of contention in debates on early gnathostome relationships. In placoderms and osteichthyans, the principal hyoid articulation is borne on a bridge or process that overlies the jugular vein (electronic supplementary material, figure S2). Conditional on the placement of placoderms as stem gnathostomes, the unusual ventral articulation of the hyoid arch is thus best interpreted as a chondrichthyan synapomorphy.

The characters presented here join a growing list of apomorphies from outside the endocranium shared by chondrichthyans and acanthodians. These include micromeric cranial and shoulder exoskeletons, lateral line canals passing between scale rows (rather than perforating or running through the scales), a dorsal endoskeletal scapular blade, median otic ridge, details of the semicircular canals and jaw articulations on the rear of the postorbital process. Furthermore, the gross resemblances of the posterior otic capsules of *Acanthodes*, *Ligulalepis* and *Pucapampella* suggest that they reflect shared primitive conditions of early gnathostomes. Consequently, short otic capsules are likely to be a primitive gnathostome condition, with the extended condition shared convergently between placoderms and some early elasmobranch-like chondrichthyans.

## 5. Conclusion

**Figure 4.**
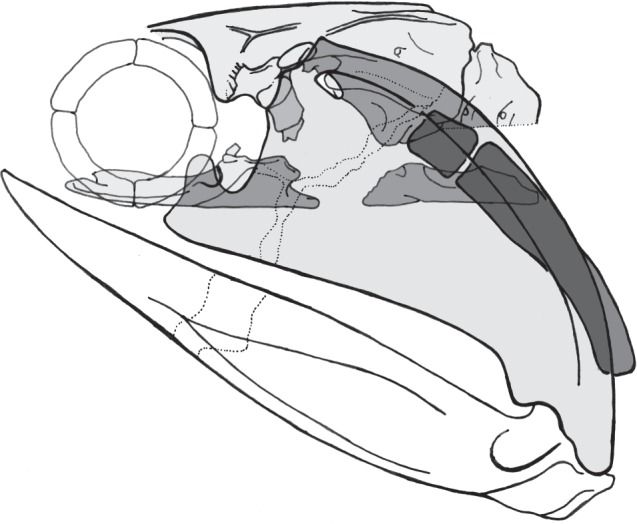
Revised reconstruction of *Acanthodes bronni* with hyoid articulation below jugular groove (adapted from [9]).

We have shown that articulation sites for the hyomandibula on the otic sidewall proposed by Miles and by Davis *et al*. are anatomically anomalous. By reference to additional specimens and comparative anatomy, we have shown that the ‘antero-lateral otic process’ of Davis *et al*.'s description is the site of the hyomandibular articulation. The result is an articulation site ventral to the course of the jugular vein (figure 4)—a clearly polarized synapomorphy of chondrichthyans.

Although early gnathostome phylogenetic relationships are currently in a state of flux, some important points of consensus are emerging between independent investigators. Most significant among these points is the shift of acanthodians to the chondrichthyan total group. The long-standing osteichthyan interpretation of *Acanthodes* has begun to fade owing to two factors: increased understanding that many of its osteichthyan-like traits are, in fact, gnathostome symplesiomorphies (such as the ventral cranial fissure [18–21]), are shared convergently with osteichthyans (e.g. ‘tropibasy’), or are simply misinterpretations. This study furthers and updates the work by Davis *et al*., which revealed that many of the chondrichthyan-like features of *Acanthodes* have been overlooked. Our observations refute the osteichthyan-like hyoid articulation reconstruction, invalidating one of the key osteichthyan-like characters of *Acanthodes* while at the same time demonstrating a shared chondrichthyan apomorphy. The chondrichthyan status for acanthodians is thus corroborated, suggesting that this revived hypothesis may indeed be a significant advance in early gnathostome phylogenetics.

## Supplementary Material

Phylogenetic data matrix and character list as nexus file

## Supplementary Material

Figure S1

## Supplementary Material

Figure S2

## Data accessibility

The stacks and all scan parameters have been uploaded to Dryad and are available for download at (http://dx.doi.org/10.5061/dryad.1h538).
