# Supplementary figures and images for: The hyoid arch and braincase anatomy of Acanthodes support chondrichthyan affinity of ‘acanthodians’
Source: Proc Biol Sci. 2015 Dec 22;282(1821):20152210. doi: 10.1098/rspb.2015.2210 (PMC4707761; doi:10.1098/rspb.2015.2210)

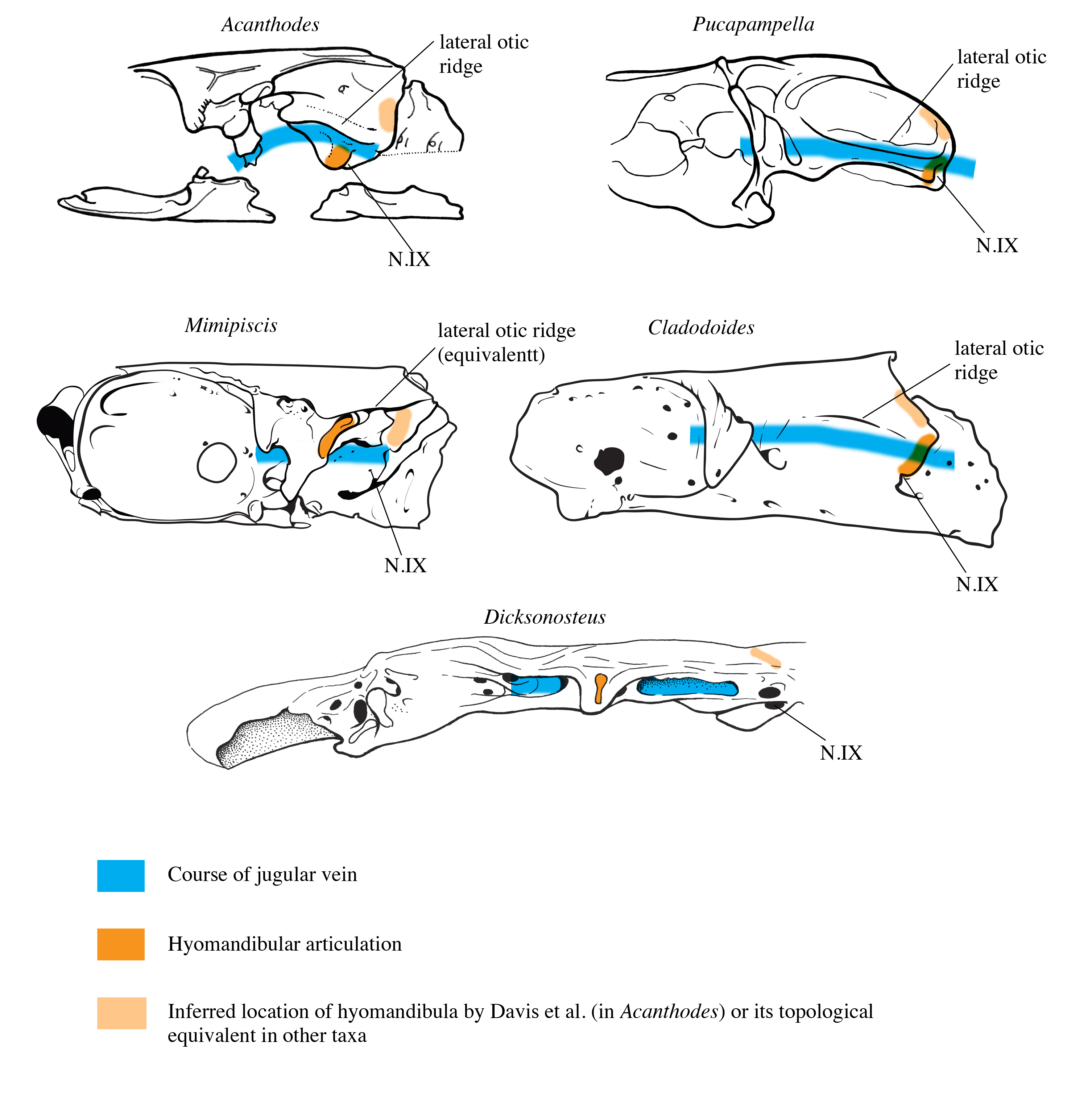

Supplement: Figure S1 [file rspb20152210supp2.jpg]

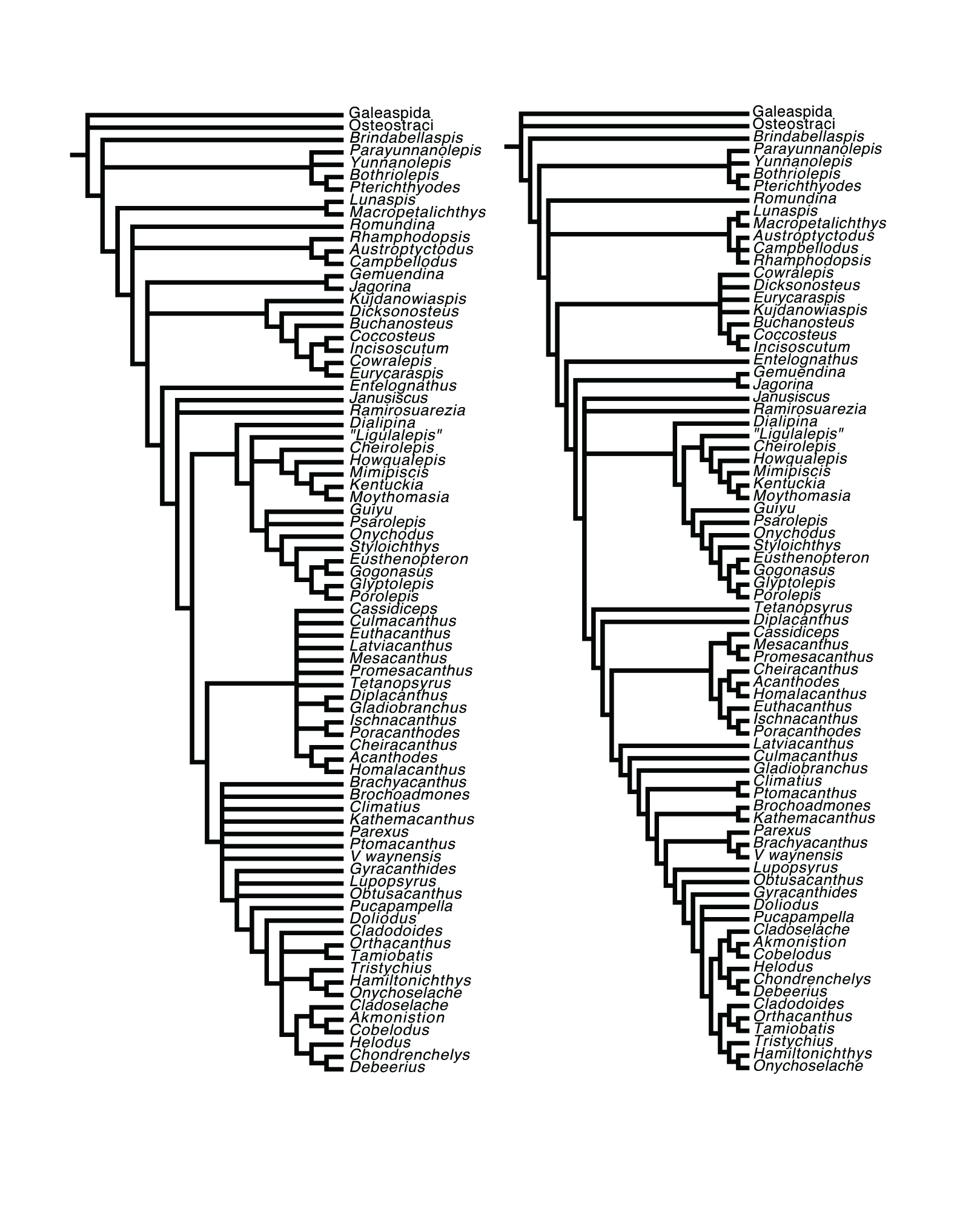

Supplement: Figure S2 [file rspb20152210supp3.tif]
